# Himalayan lichen biomass for green synthesis of silver nanocolloids: growth kinetics, effect of pH and metal sensing

**DOI:** 10.1098/rsos.231633

**Published:** 2024-03-06

**Authors:** Nirmala Sharma, Surendra Kumar Gautam, Achyut Adhikari, Bhanu Bhakta Neupane

**Affiliations:** ^1^ Central Department of Chemistry, Tribhuvan University, Kirtipur, Kathmandu 44613, Nepal; ^2^ Department of Chemistry, Tri-Chandra Multiple Campus, Tribhuvan University, Kathmandu 44605, Nepal

**Keywords:** growth kinetics, heavy metals, materials science, nanoparticles, surface plasmon resonance

## Abstract

Lichen is one of the most abundant non-vascular biomasses; however, a systematic study on the application of biomass in nanomaterial synthesis is very limited. In this study, an aqueous lichen extract was obtained from *Hypotrachyna cirrhata*, one of the most abundant Himalayan lichen biomasses, using a simple cold percolation method. The effects of extract-to-silver nitrate mixing ratio, pH and waiting time on the growth and stability of nanoparticles were systematically explored. The rate constant for bio-reduction was found to be 5.3 × 10^−3^ min^−1^. Transmission electron microscopy showed a narrow particle size distribution with a mean particle size of 11.1 ± 3.6 nm (*n* = 200). The X-ray diffraction and selected area electron diffraction techniques confirmed the formation of cubic crystals. The synthesized colloidal solution showed excellent response to Hg^2+^ and Cu^2+^ ions in spiked water samples. The limit of detection and calibration sensitivity for Hg^2+^ and Cu^2+^ ions were found to be 1 and 5 mg l^−1^ and 2.9 × 10^−3^ and 1.6 × 10^−3^ units ppm^−1^, respectively. These findings suggested that spherical silver nanoparticles with a narrow particle size distribution can be synthesized on a laboratory scale using an aqueous *H. cirrhata* lichen extract, and the colloidal solution can be used for the detection of selected heavy metals in water samples.

## 1. Introduction

Nanostructured materials have individual domains or units in the size range of 1–100 nm. In recent decades, such materials have been of interest mostly in physics, chemistry, biology and engineering disciplines. Nanomaterials of the size range of 1–20 nm are of special interest as they offer unique physico-chemical properties such as high surface-to-volume ratio and high diffusion rates. They are widely explored for biomedical [1], optoelectronic [2,3], catalytic [4,5], sensing and several other applications [6]. Nanomaterials having diverse chemical composition and morphology are being explored. Based on morphology, nanomaterials can be broadly classified into three-dimensional, two-dimensional, one-dimensional and zero-dimensional [7]. Particles having diverse morphology, such as nanospheres [8], nanorods [9], nanoclusters [10], nanodots [11], nanowires, nanosheets [12], nanocubes and nanoboxes [13], are reported. Such materials are also finding applications in cosmetics [14], healthcare [15,16], food industries [15,17] and environmental remediation [18].

Several methods for nanomaterial synthesis are available, and these methods can be broadly classified into physical, chemical and biological methods [19]. Although physical methods, such as the chemical vapour deposition technique, avoid solvent contamination and offer large-scale production, they require sophisticated instrumentation and high energy for evaporation and condensation of particles, and normally result in broad size distribution. In chemical techniques, suitable reducing and capping agents are used to synthesize nanoparticles with a narrow particle size distribution. Although traditional wet methods normally do not require sophisticated instrumentation, toxic precursors and small-scale production are the limitations. In biological methods, the precursors needed for the reduction and stabilization are obtained from microorganisms (fungi and bacteria), plant extracts and metabolites [19–29]. Although size control and small-scale production are challenges, the method is being explored as a cost-effective and eco-friendly method for nanoparticle synthesis [20,30].

The spherical nanoparticles of silver, gold and copper are of special interest due to their low toxicity, high stability and ease of synthesis and functionalization for biomedical and sensing applications [8,16,20,31,32]. In recent decades, these particles have been synthesized on a laboratory scale using crude and/or partially purified extracts and metabolites obtained from the root, leaf, stem, flower and fruit parts of various plants [19–24]. The extract obtained from algae, fungi and mushrooms is also being explored for green synthesis [25,26,33–36]. However, to get nanoparticles of stable size and shape, several parameters, such as composition, metal salt-to-extract ratio, waiting time and pH, have to be controlled.

Lichen biomass is one of the most abundant non-vascular biomasses on the earth’s surface. The biomass is found on tree trunks and branches. The degree of colonization and distribution is determined by host attributes and climatic variation [37]. The biomass is found useful in making dyes and perfumes, as food, in traditional medicine and as bio-indicators [38,39]. Applications of lichen biomass in the green synthesis of metallic nanoparticles are also reported in many studies [40–46]. The majority of the studies are focused on antimicrobial applications. Therefore, it would be interesting to explore the heavy metal-sensing potential of nanoparticles.

In this study, the application of the aqueous extract obtained from *Hypotrachyna cirrhata*, one of the most abundant high-altitude lichen species in the Himalayan region of Nepal, is systematically explored for the synthesis of silver nanoparticles (AgNPs). The effects of extract-to-metal salt ratio, pH and waiting time on growth kinetics and stability of AgNPs are systematically explored. The synthesized nanoparticles are characterized using UV–visible (Vis), X-ray diffraction (XRD), scanning electron microscopy (SEM) and transmission electron microscopy (TEM) techniques. Finally, the sensing potential of the colloidal solution for 10 metal ions in spiked water samples is explored.

## 2. Experimental section

### 2.1. Materials

**Figure 1.**
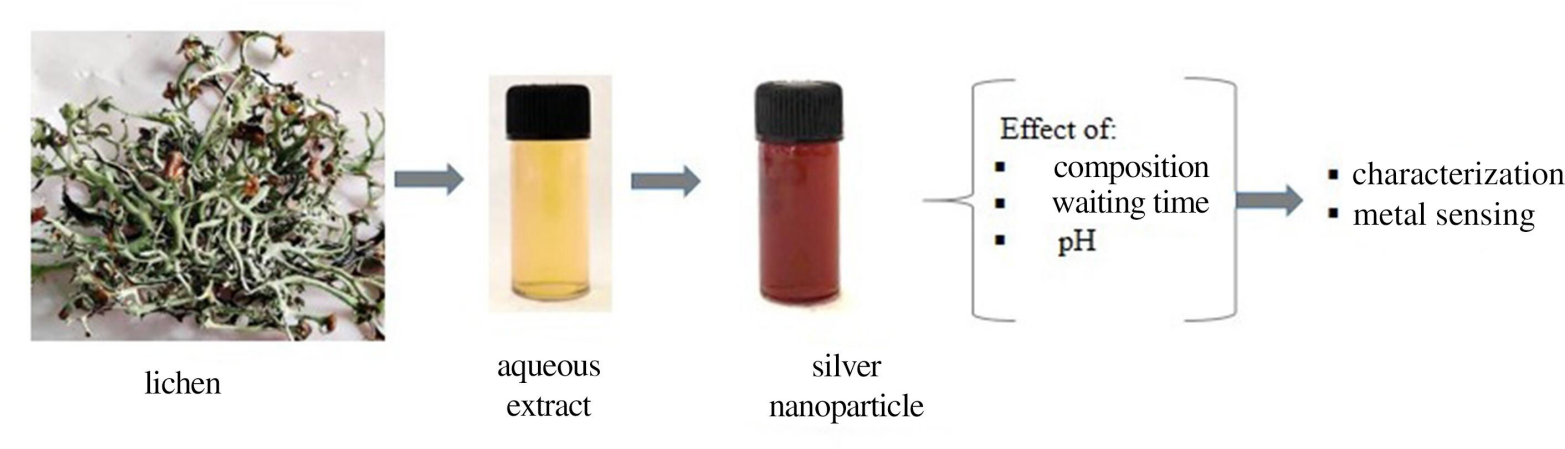
Experimental design. The major steps involved in this research along with a representative photograph of *Hypotrachyna cirrhata* lichen species.

Lichen biomass was collected from an altitude of 2300 m in Daman (latitude 27.6081833 and longitude 85.0922813), Makwanpur district of central Nepal. The lichen species were identified to be *H. cirrhata (Fr*.) Divakar, A. Crespo, Sipman, Elix and Lumbsch (figure 1) in the National Herbarium and Plant Laboratories, Department of Plant Resources, Ministry of Forests and Environment, Nepal. Permission to harvest lichen for the scientific study was obtained from the Department of Plant Resources, Ministry of Forests and Environment, Nepal (letter number 2076/77-264).

The biomass was rinsed adequately with running tap water, followed by distilled water, and then dried at room temperature in the shade for 2 weeks. An electrical blender was used to grind the biomass into powder and then stored in polyethylene bags for further use. The stock lichen extract solution was prepared by adding 2 g of lichen powder to 100 ml of distilled water. The content was mixed using a magnetic stirrer at 90°C for 30 min, cooled and filtered through a Whatman filter paper (WHA7404004, 47 mm diameter and 0.45 μm pore size). The filtrate was stored at 4°C in the dark for further experiments.

### 2.2. Biosynthesis of silver nanoparticles

A stock solution of 50 mM AgNO_3_ was prepared by dissolving 4.25 g of AgNO_3_ in 500 ml distilled water in a volumetric flask. Next, 10 ml of the stock solution was diluted to 500 ml to prepare 1 mM of AgNO_3_ to synthesize AgNPs. The lichen extract was then mixed with the silver nitrate solution in different volume ratios while being mixed using a magnetic stirrer at room temperature. The visual change in colour of the solution from yellow to dark brown was noted.

The kinetics of bio-reduction was monitored by recording surface plasmon resonance (SPR) spectra in the range of 300–700 nm (UV-1900 spectrophotometer; Shimadzu) from shorter to longer time at a fixed pH and silver nitrate:lichen extract volume ratio. The effects of pH on the SPR spectra were investigated by varying the pH of the growth solution from 2 to 12 using a 0.1 M NaOH solution while maintaining the composition of silver nitrate and extract constant.

### 2.3. Characterization of nanoparticles

The zeta potential was measured using a zeta sizer (SZ-100; HORIBA Scientific). Before measurement, particles were dispersed in distilled water and sonicated for 10 min. The scattering angle, sample holder temperature and dispersion medium viscosity were 90^o^, 25.0°C and 0.896 mPas, respectively.

The Fourier-transform infrared spectroscopy (FTIR) measurement of the lichen extract was taken in an attenuated total reflection mode (Nicolet; Thermo Fisher Scientific) in the range of 400–4000 cm^−1^. The spectral resolution and the number of scans during the measurement were set to 4 cm^−1^ and 100, respectively.

For XRD measurement, the nanoparticle solution was centrifuged at 9000 r.p.m. (Sorvall ST 8R centrifuge) for 20 min at 25°C. The supernatant was discarded, and the pellet was re-dispersed in distilled water. The centrifugation process was repeated three times to wash off any adsorbed substance on the surface of AgNPs and finally washed with absolute ethanol. The content was dried at room temperature and stored in an Eppendorf tube covered with aluminium foil. The XRD data of the nanopowder were collected using an X-ray diffractometer (MiniFlex 600; Rigaku), consisting of CuKα (λ = 1.540Å) as an X-ray source. The step size, scanning range and scanning speed were set to 0.05^o^, 20‒90^o^ and 0.25^o^ s^−1^, respectively.

SEM images and energy-dispersive X-ray (EDX) spectra were measured with a field-emission scanning electron microscope (JEOL). The particles were dispersed on a carbon tape and sputter coated with Au. For TEM analysis, the colloidal suspension was deposited on a copper grid. TEM images and selected area diffraction or selected area electron diffraction (SAED) patterns were measured using a TEM microscope (JEM-2100Plus; JEOL). The TEM images were analysed in ImageJ software (National Institues of Health, USA) to get information on particle size distribution.

### 2.4. Metal ion sensing

In a regular sensing test, 1 ml of the metal solution was added to 4 ml of the AgNP solution. The ions used for the tests were Fe^2+^, Ba^2+^, Hg^2+^, Cu^2+^, Mn^2+^, Zn^2+^, As^3+^, Ni^2+^, Cr^3+^ and Cd^2+^ at a concentration of 2.5 × 10^−4^ M. The colour change in the mixture was observed visually, and the SPR spectra were also measured to see the change in peak intensity, position and shape. The metal ions that showed a drastic change in SPR were spiked into the nanoparticle suspension to see the systematic change in SPR spectra. The spectra were analysed to get information on the limit of detection and the calibration sensitivity.

A schematic diagram that depicts the overall experimental design used in this work is provided in figure 1.

## 3. Results and discussion

### 3.1. Effect of precursor concentration

**Figure 2.**
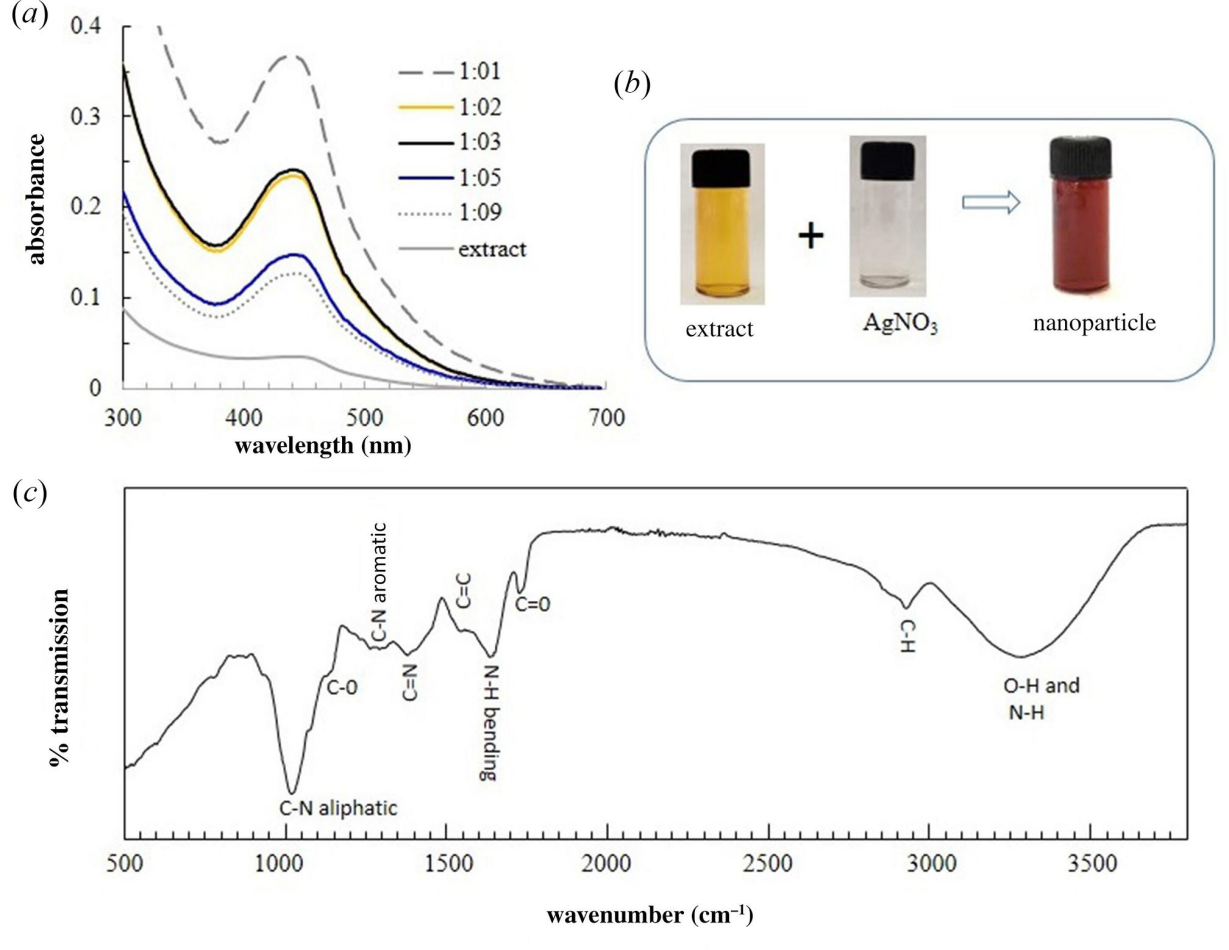
UV–Vis and FTIR data. (*a*) UV–Vis spectra recorded at a variable extract-to-silver nitrate (1 mM) volume ratio. (*b*) A visual change in colour on mixing the extract and silver nitrate in a 1:1 volume ratio. (*c*) FTIR spectra of aqueous extract powder.

The growth of nanoparticles depends on several parameters such as extract-to-metal ion ratio, waiting time and pH. To explore the effect of volume ratios in the formation of nanoparticles, UV–Vis spectral change was recorded after a fixed waiting time of 24 h while varying the silver nitrate and lichen extract volume ratios from 1:1 to 1:9 (figure 2*a*). The pH in all cases was ~7.5. The spectral feature peaking at ~435 nm in all volume ratios is the SPR band. The feature provides strong evidence for AgNP formation. Other evidence is provided by the development of reddish-brown colouration on mixing a light yellowish extract solution with a colourless silver nitrate solution (figure 1*b* ).

The pH of the mixture was ~7.5 in all cases, so the difference in spectral shape (figure 2*a*) is mainly attributed to compositional variation. A well-defined and intense SPR was observed when the extract-to-silver nitrate volume ratio was 1:1. The SPR band is also symmetric. This could suggest that at a 1:1 volume ratio, AgNPs having a narrower particle size distribution could have formed. Direct evidence for the particle size distribution can be obtained from transmission electron microscopic images reported in the later section.

The nanoparticle growth kinetics could involve reduction of Ag^+^ to Ag^0^ state, formation of silver nuclei and the growth of nuclei to AgNPs. The reduction could be due to electron-donating moieties, such as -OH and -NH_2_ groups present in the organic precursors, or phytochemicals, such as polyphenols and proteins. The organic precursors could also help in capping and stabilization of nanoparticles [20–22,30,47].

Preliminary information on the presence of different precursors involved in bio-reduction can be obtained from the FTIR spectra of the extract (figure 2*c*). A broad and asymmetric band in the range of 3000–3600 cm^−1^ and peak ~3300 cm^−1^ can have mixed contributions from the stretching vibration of phenolic -O–H and protein -N–H. The bands observed at ~3400 and 2920 cm^−1^ can be assigned to the -C–H stretching vibrations of the primary and secondary amines, respectively. The band at ~1730 cm^−1^ could correspond to the -C=O stretching frequency of carboxylic anhydrides, ketones or lactones [48]. The bands at ~3400 and 1640 cm^−1^ can be attributed to -N–H stretching and bending vibrations in amines in proteins [49]. The band at ~2920 cm^−1^ could arise from C–H stretching, and at ~1530 cm^−1^ corresponds to C=C stretching vibration from aromatic rings of plant metabolites [21]. The peak at 1030 cm^−1^ can arise from either =C–H in-plane bending or -C–N stretching of aliphatic amines, and the band at ~1390 cm^−1^ arises from C=N stretching; the band at ~1140 cm^−1^ arises from C−O stretching phenol, ester or ether groups and the band at 1260 cm^−1^ arises from C–N stretching aromatic amines [48,50,51]. These signatures indicate that polyphenols, proteins or other plant metabolites present in lichen extract could be responsible for the reduction, capping and stabilization of the AgNPs.

### 3.2. Growth kinetics

**Figure 3.**
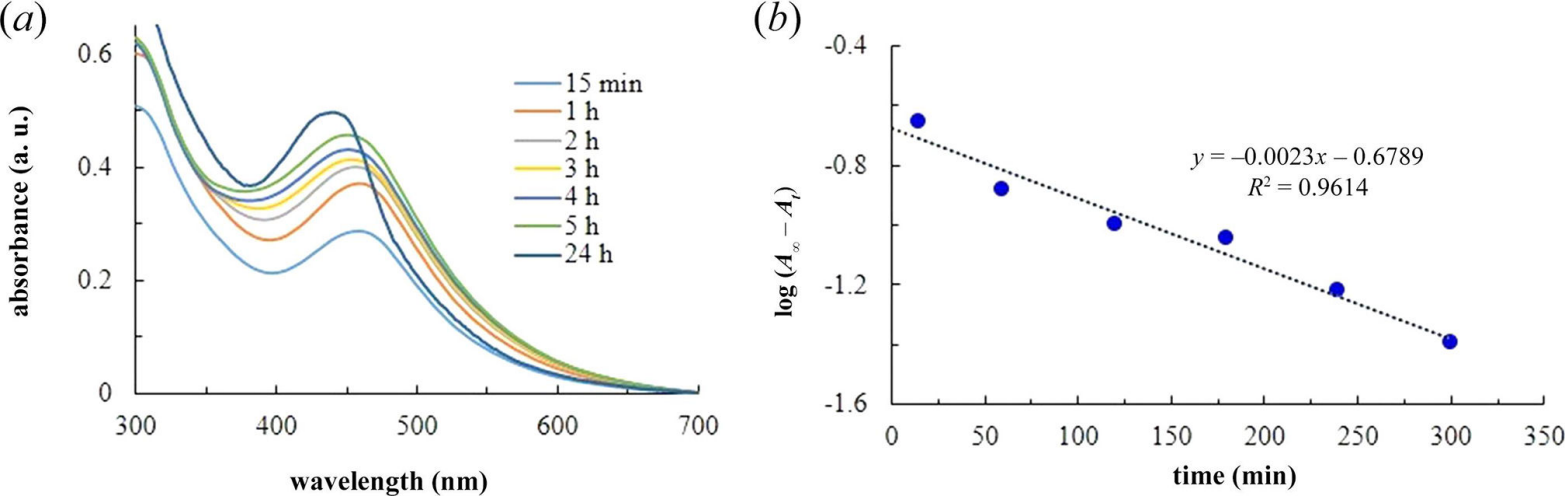
Growth kinetics. (*a*) UV–Vis spectra of AgNP recorded at different waiting times. (*b*) The experimental kinetic data (blue dots) and the linear fit to the data (dotted lines).

At fixed precursor ratios and pH, SPR spectra were recorded from shorter to longer waiting times (figure 3*a*). As expected, the SPR peak intensity increases with waiting times. A small shift in peak position could be due to a change in particle size during nucleation and growth of nanoparticles.

The SPR spectra were further analysed to get information on the rate constant (*k*) for bio-reduction, considering a first-order kinetic model (equation (3.1)).


(3.1) $$log_{10}(A_{\infty }-A_{t})=log_{10}(A_{\infty }-A_{0})\;-\frac{kt}{2.303},$$

where *A_∞_*, *A_t_* and *A*_0_ are the SPR peak intensities at infinite waiting times (here 24 h), at time *t* and at zero time, respectively. Equation (3.1) suggests that the slope of log(*A_∞_ − A_t_*) versus *t* plot provides information on the rate constant. Indeed, the first-order kinetic model fits well (*R*^2^ > 0.95) with the experimental data (figure 3*b*). The rate constant was found to be 5.3 × 10^−3^ min^−1^.

### 3.3. Effect of pH

**Figure 4.**
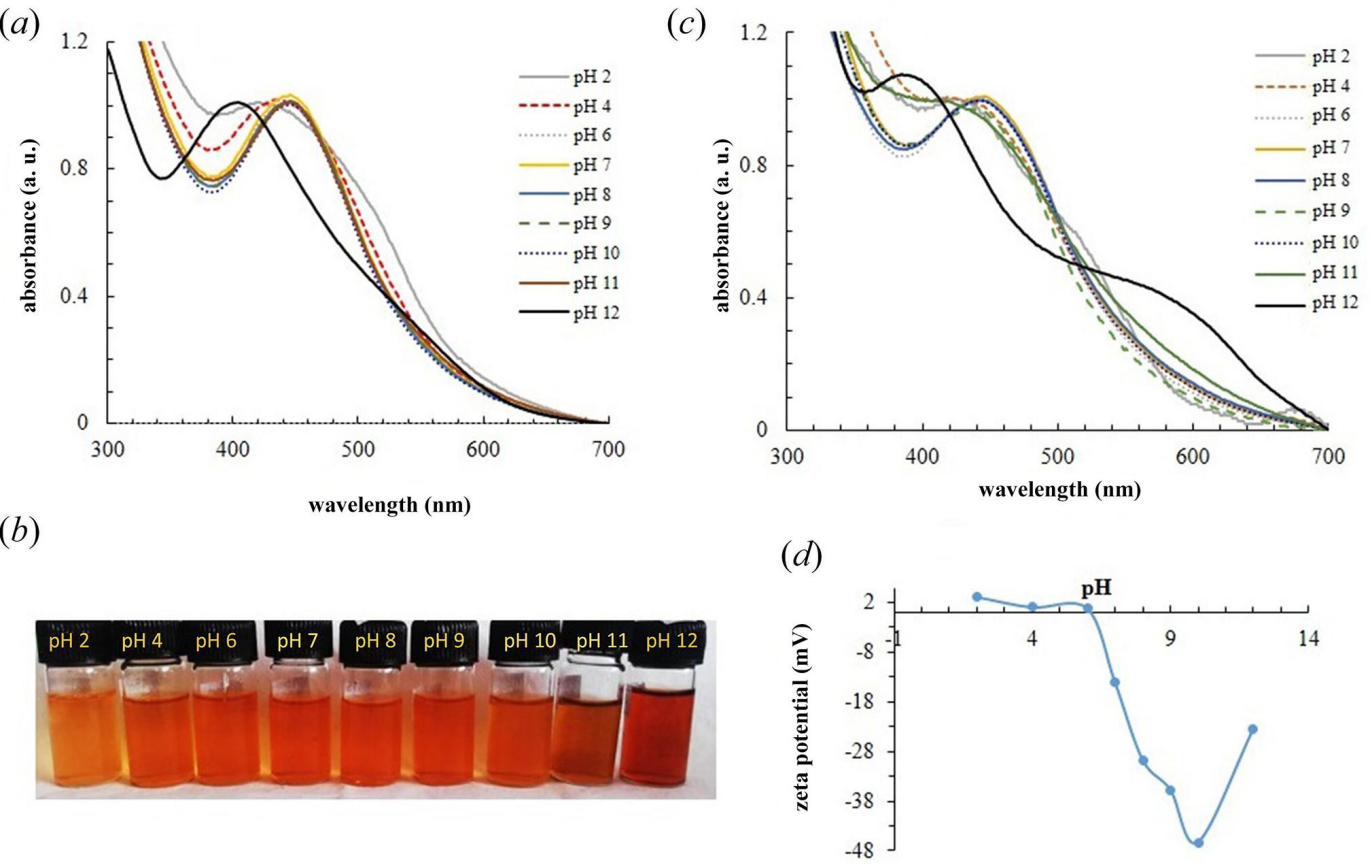
Effects of pH on the SPR spectra. (*a*) SPR spectral profiles at pH 2–12 measured after 24 h. (*b*) The corresponding visual colour change. (*c*) SPR spectra measured after 4 weeks. For easy comparison, all SPR spectra are normalized to the SPR peak. (*d*) Zeta potential measured at different pH values.

To explore the effect of pH, UV–Vis spectra were recorded in the pH range of 2–12 at the fixed waiting time of 24 h (figure 4*a*). The corresponding visual colour change was also recorded at all pH ranges (figure 4*b*). The SPR spectra were also measured at a longer waiting time of 4 weeks to explore the stability of the nanoparticle solution (figure 4*c*).

At a shorter waiting time and a pH range of 4–11, a well-defined and symmetric SPR band is visible (figure 4*a*). At pH 2 and 12, the SPR band becomes more asymmetric with a shoulder of ~550 nm. The additional feature could be due to aggregation of nanoparticles. At a longer waiting time of 4 weeks (figure 4*c*) and a pH range of 4–11, the SPR spectral shape is unchanged. However, at pH of 2 and 12, the 550 nm feature becomes more pronounced. This could be due to an increase in the population of aggregated particles.

To gain more insight into the stability of colloidal solution, zeta potential values were measured. The zeta potential is a direct measure of the surface charge density. Colloidal particles having high zeta potential (both positive and negative values) tend to be stable, i.e. do not aggregate and flocculate, due to electrostatic repulsion. The zeta potential of AgNPs is slightly positive at low pH values (2, 4 and 6) and flips to negative values at pH > 7. The zeta potential is highest at pH 10. This could suggest that the particles remain most stable at this pH. For long-term stability, the zeta potential should remain stable over time. At pH 12, the zeta potential is significantly high. The particle aggregation at a longer waiting time, as indicated by the appearance of an additional band ~550–600 nm (figure 4*c*), could be due to unable charge density, i.e. decrease in zeta potential over time.

### 3.4. SEM and XRD measurements

**Figure 5.**
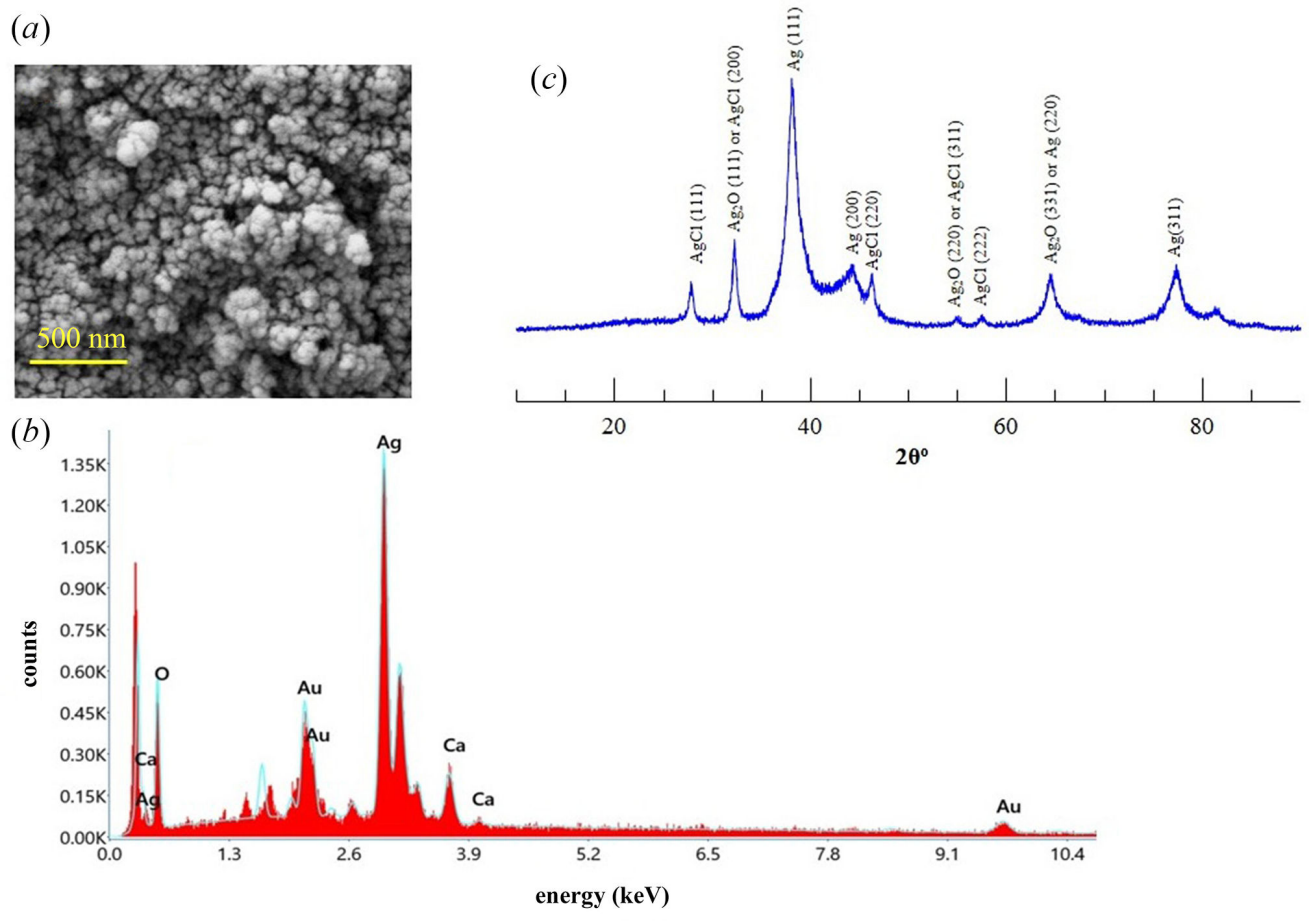
SEM, SEM-EDX and XRD data. (*a, b*) SEM and EDX data. A scale bar of 500 nm is shown in (*a*). (*c*) XRD data for the AgNP. The numbers given in parentheses indicate the major diffraction planes corresponding to Ag and AgCl/Ag_2_O nanoparticles.

The nanoparticle agglomerates or aggregates are visible in SEM images (figure 5*a*). Therefore, individual particle size information is difficult to obtain from the image. The agglomerate formation is a result of high surface energy and is commonly reported in SEM images of AgNPs synthesized using other plant extracts [48,51]. As expected, the SEM-EDX data showed distinct peak characteristics of silver (figure 5*b*). The additional peaks could come from impurities and Au used in the sample preparation.

In XRD data (figure 5*c*), the peaks at 2*θ* values of 38.08^o^, 44.2^o^, 64.7^o^ and 77.35° can be assigned to (111), (200), (220) and (311) diffraction planes of face-centred cubic structures of AgNPs [48,51,52]. The minor peaks at 27.95^o^, 32.28^o^, 46.2^o^, 55.12^o^, 57.2^o^ and 81.5^o^ could originate from Ag_2_O and/or AgCl nanoparticles [50,53,54] and are also reported in other studies [21,51]. These peaks might originate due to the oxidation of AgNPs during XRD sample preparation and measurement.

The XRD peak characteristics of the AgNPs were used to get information on inter-planar spacing (*d_hkl_*) and average crystallite size (*L*) (table 1). The *d_hkl_* was calculated using Bragg’s equation:

**Table 1.** Crystallite size and lattice parameters from XRD data.

	**2*θ* ^o^**	***hkl***	***d*_*hkl*_ (Å)**	***β* (rad)**	**crystallite size (nm)**	**mean ± s.d. (nm)**
AgNP	38.08	(111)	2.36	0.0192	7.6	7.3 ± 0.5
	46.2	(200)	2.00	—	—	
	64.48	(220)	1.44	0.0216	7.5	
	77.35	(311)	1.23	0.0267	6.6	


(3.2) $$n\lambda =2d_{hkl}\;\sin\theta ,$$

where *n* = 1 (first-order diffraction), *λ* is the wavelength of the X-ray source (0.154 nm) and *θ* is the peak position (in radians).

The crystallite size (*L*) was calculated using the Debye–Scherrer equation [55].


(3.3) $$L=\frac{0.9\lambda }{\beta \cos\theta },$$

where *λ* is the wavelength of the X-ray source (here 0.154 nm), *β* is the full width at a half maximum of each diffraction peak (in radians) and *θ* is the peak position (in radians). The *β* factor was measured using three major peaks at 38.08^o^, 64.7^o^ and 77.35^o^. The average crystallite size was found to be 7.3 ± 0.5 nm (table 1).

### 3.5. TEM imaging

**Figure 6.**
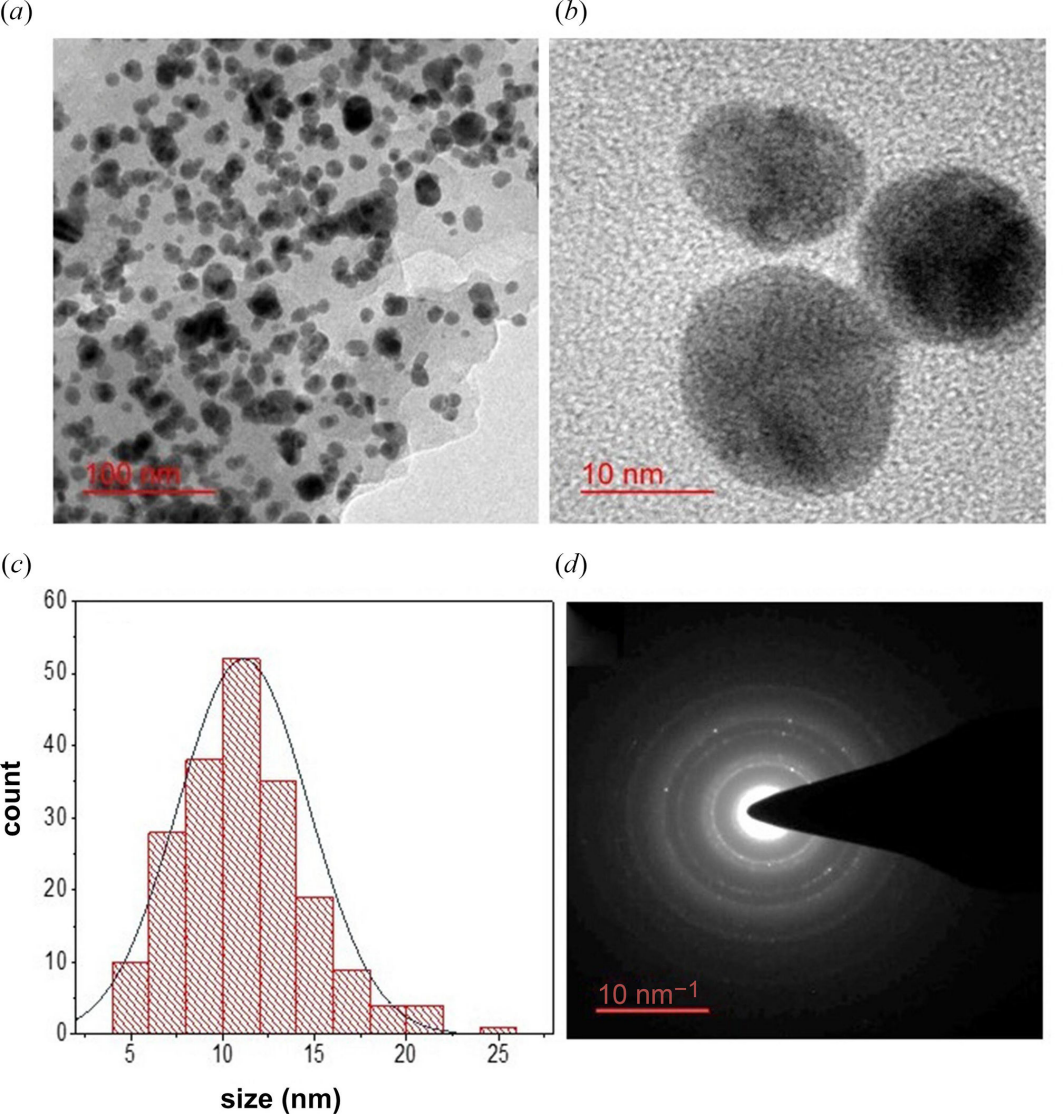
TEM images and SAED patterns. (*a*, *b*) TEM images measured at lower and higher magnifications, respectively. A scale bar of 100 and 10 nm is shown in (*a*) and (*b*), respectively. (*c*) A histogram plot (*n* = 200 and bin size of 2 nm) to show the particle size distribution. The solid curve is the normal/Gaussian fit to the histogram plot. (*d*) The SAED pattern. A scale bar of 10 nm^−1^ is shown.

TEM is the most important technique to get morphological information on metallic nanoparticles. TEM images (figure 6*a*,*b*) show individual nanoparticles of spherical shape. The mean particle size obtained from the image analysis (*n* = 200) at 95% confidence interval was found to be 11.1 ± 0.5 nm and the particle size ranged from 4.2 to 29 nm. To show the particle size distribution, a histogram plot of 200 particles is also shown (figure 6*c*). Interestingly, the histogram can be fitted well with a narrow Gaussian curve, suggesting that particles are monodispersed. This observation is consistent with a symmetric SPR band observed in the UV–Vis spectra.

The SAED pattern of the nanoparticle sample was also measured to get further information on crystallinity. The SAED pattern showed four distinct and sharp concentric rings (figure 6*d*). These rings indicate that particles are highly crystalline. The SAED patterns were analysed to get inter-planar spacing (*d_hkl_*) in Å following equation (3.4).


(3.4) $$d_{hkl}=10{\left(\frac{a}{2}\right)}^{-1},$$

where *a* is the diameter of each concentric ring in nm^−1^. The inter-planar spacing (*d_hkl_*) was found to be 1.23Å, 2.35Å, 2.04Å, 1.41Å and 1.22Å. Interestingly, *d_hkl_* values obtained from SAED and XRD perfectly correlate (*r* = +0.99).

### 3.6. Calorimetric metal sensing

**Figure 7.**
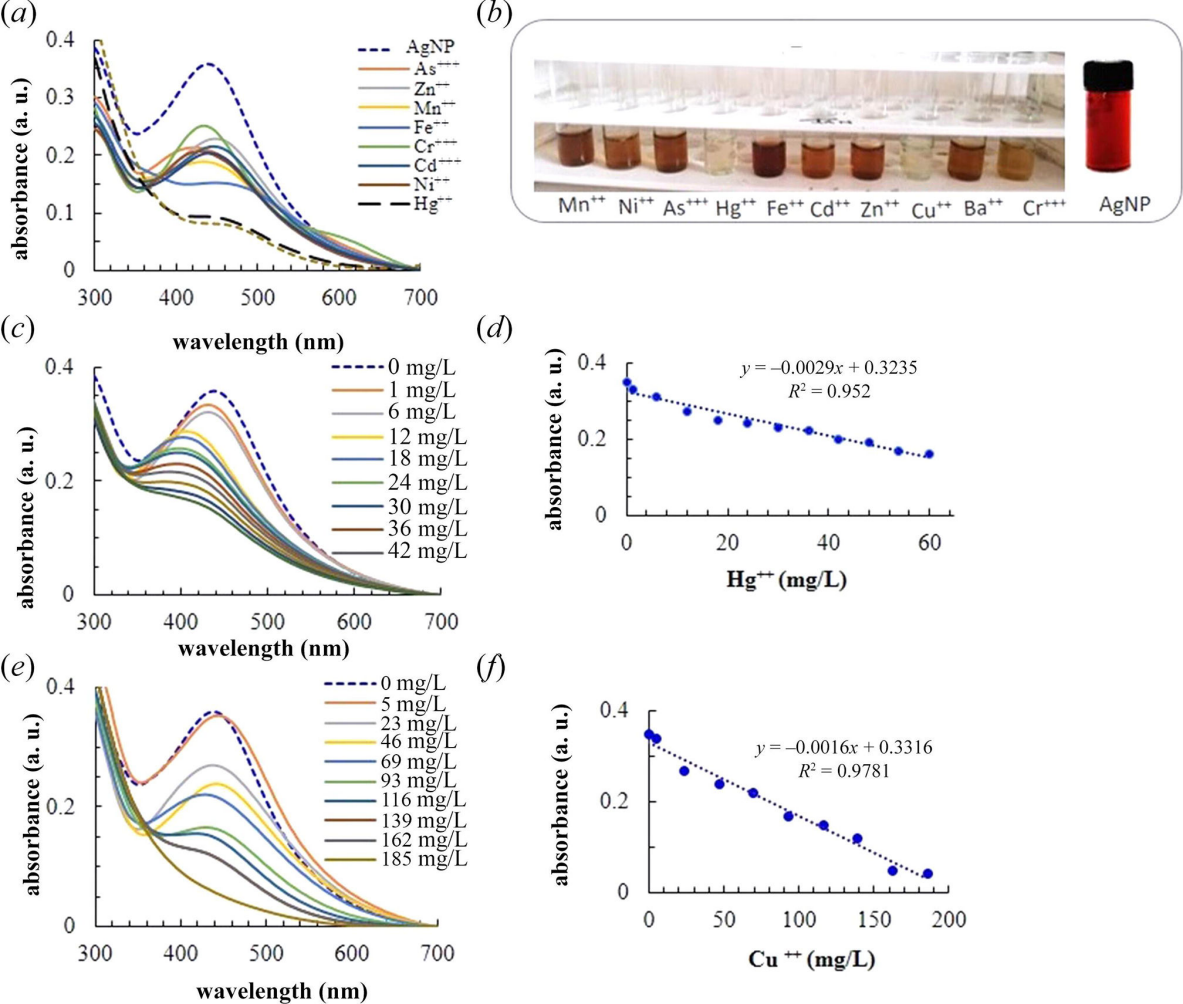
Calorimetric sensing of ions. (*a*) UV–Vis spectra of AgNP dispersion spiked with a fixed concentration of different metal ions. (*b*) The photograph of glass tubes filled with a nanoparticle solution spiked with different ions. The tube holder could accommodate only 10 tubes. Therefore, a photograph of the blank (unspiked) nanoparticle solution (labelled AgNP) filled in a glass vial is also shown. The photos were taken under similar light and contrast conditions. (*c*) SPR spectra recorded at a variable Hg^2+^ concentration and (*d*) the corresponding intensity versus concentration plot. (*e*) SPR spectra recorded at a variable Cu^2+^ concentration and (*f*) the corresponding intensity versus concentration plot. The dotted lines in (*e*) and (*f*) show the linear regression to the experimental data.

The calorimetric sensing potential of AgNP was tested for 10 heavy metals and trace elements. For preliminary screening, nanoparticle solution was spiked at a 2.5 × 10^−4^ M concentration of Fe^2+^, Ba^2+^, Hg^2+^, Cu^2+^, Mn^2+^, Zn^2+^, As^3+^, Ni^2+^, Cr^3+^ and Cd^2+^. The change in the SPR profile and the visual colour was recorded (figure 7*a*,*b*).

The SPR peak shows an ion-specific response. A decrease in peak intensity is observed in the presence of all ions. Additionally, the SPR band shifts either to blue or to red, with an increase in the bandwidth. For example, in the presence of Hg^2+^ and Cu^2+^, the SPR band almost disappears, and As^3+^ decreases the peak intensity along with the appearance of one additional band at ~600 nm (figure 7*a*). In a broader sense, the change in the spectral feature is due to the interaction of foreign ions with the biomolecule adsorbed on the surface of nanoparticles, which can lead to nanoparticle aggregation [56]. The spectral change also correlates with the colour change of the nanoparticle solution (figure 7*b*). Particularly, the addition of Hg^2+^ and Cu^2+^ converted the reddish-brown-coloured AgNP solution into a completely colourless solution. These observations suggest that the SPR band shows the maximum response with Cu^2+^ and Hg^2+^ ions.

The reduction potential of Hg^2+^/Hg is higher than that of Ag^+^/Ag system (E^0^_Hg_^2+^/_Hg_ = +0.85 V and E^0^_Ag_^+^_/Ag_ = +0.80 V). Therefore, a redox reaction is possible with the formation of metallic mercury. The newly formed metallic mercury could interact strongly with Ag, leading to the formation of amalgam on the surface of AgNPs. This could lead to the damping of SPR [21,57]. Similar reasoning could be applied to Cu^2+^.

To quantify the sensing performance, the nanoparticle solution was titrated with variable concentrations of Hg^2+^ and Cu^2+^, and a change in the SPR profile was recorded (figure 7*c,e*). The intensity change showed a linear response with the concentration of both ions (figure 7*d,f*). The calibration sensitivity as determined from the slope of the respective curves for Hg^2+^ and Cu^2+^ was found to be 1.6 × 10^−3^ and 2.9 × 10^−3^ units ppm^−1^, respectively. The limit of detection for Hg^2+^ and Cu^2+^ ions was found to be 1 and 5 mg l^−1^, respectively. These findings suggest that green synthesis of spherical AgNPs is possible using aqueous lichen extract, and nanoparticles can be used for the detection of selected heavy metals.

## 4. Conclusions

In this study, the application of the aqueous lichen extract obtained from the high-altitude lichen species *H. cirrhata* was systematically explored for the synthesis of AgNPs. The most intense and sharp SPR band at ~435 nm was obtained in the UV–Vis spectra when the extract-to-Ag^+^ ratio was 1:1 (v/v) and at a pH range of 9–10. The nanocolloidal solution showed maximum stability at a pH range of 8–11, which is consistent with the high zeta potential in the pH range. The rate constant for bio-reduction was found to be 5.3 × 10^−3^ min^−1^. The mean particle size (*n* = 200) obtained from TEM was found to be 11.1 ± 3.6 nm. The SAED and XRD data indicated the formation of cubic crystals. The nanocolloidal solution showed excellent sensitivity for the presence of Hg^2+^ and Cu^2+^ ions in spiked water samples. The limit of detection and calibration sensitivity for ions were found to be 1 and 5 mg l^−1^ and 1.6 × 10^−3^ and 2.9 × 10^−3^ units ppm^−1^, respectively. These findings suggested that green synthesis of spherical AgNPs having a narrow particle size distribution is possible using the aqueous lichen extract of *H. cirrhata,* and the colloidal solution can be used for the detection of selected heavy metals.

## Data Availability

The datasets are provided as electronic supplementary material [58].
